# Are Body Composition Parameters Better than Conventional Anthropometric Measures in Predicting Pediatric Hypertension?

**DOI:** 10.3390/ijerph17165771

**Published:** 2020-08-10

**Authors:** Chih-Yu Hsu, Rong-Ho Lin, Yu-Ching Lin, Jau-Yuan Chen, Wen-Cheng Li, Li-Ang Lee, Keng-Hao Liu, Hai-Hua Chuang

**Affiliations:** 1 Department of Family Medicine, Chang Gung Memorial Hospital, Linkou and Taipei Branches, Taoyuan 33305, Taiwan; b85079@gmail.com (C.-Y.H.); welins@cgmh.org.tw (J.-Y.C.); 620313@cgmh.org.tw (W.-C.L.); 2 Department of Industrial Engineering and Management, National Taipei University of Technology, Taipei 10608, Taiwan; rhlin@mail.ntut.edu.tw; 3 College of Medicine, Chang Gung University, Taoyuan 33302, Taiwan; yuching1221@gmail.com (Y.-C.L.); 5738@cgmh.org.tw (L.-A.L.); 4 Department of Imaging and Intervention, Chang Gang Memorial Hospital, Keelung Branch, Keelung 20401, Taiwan; 5 Department of Otorhinolaryngology—Head and Neck Surgery, Chang Gung Memorial Hospital, Linkou Branch, Taoyuan 33305, Taiwan; 6 Sleep Center, Chang Gung Memorial Hospital, Linkou Branch, Taoyuan 33305, Taiwan; 7 Department of General Surgery, Chang Gung Memorial Hospital, Linkou Branch, Taoyuan 33305, Taiwan; kenghao@cgmh.org.tw; 8 Obesity Institute & Genomic Medicine Institute, Geisinger, Danville, PA 17837, USA

**Keywords:** body-fat percentage, body mass index, children, fat mass, fat-free mass, hypertension, muscle weight, waist-to-height ratio

## Abstract

Body composition (BC) parameters are associated with cardiometabolic diseases in children; however, the importance of BC parameters for predicting pediatric hypertension is inconclusive. This cross-sectional study aimed to compare the difference in predictive values of BC parameters and conventional anthropometric measures for pediatric hypertension in school-aged children. A total of 340 children (177 girls and 163 boys) with a mean age of 8.8 ± 1.7 years and mean body mass index (BMI) z-score of 0.50 ± 1.24 were enrolled (102 hypertensive children and 238 normotensive children). Significantly higher values of anthropometric measures (BMI, BMI z-score, BMI percentile, waist-to-height ratio) and BC parameters (body-fat percentage, muscle weight, fat mass, fat-free mass) were observed among the hypertensive subgroup compared to their normotensive counterparts. A prediction model combining fat mass ≥ 3.65 kg and fat-free mass ≥ 34.65 kg (area under the receiver operating characteristic curve = 0.688; sensitivity = 66.7%; specificity = 89.9%) performed better than BMI alone (area under the receiver operating characteristic curve = 0.649; sensitivity = 55.9%; specificity = 73.9%) in predicting hypertension. In conclusion, BC parameters are better than anthropometric measures in predicting pediatric hypertension. BC measuring is a reasonable approach for risk stratification in pediatric hypertension.

## 1. Introduction

The prevalence of pediatric hypertension in school-aged children is estimated to be 0.3–4.5%, with risk factors including overweight or obesity, male sex, older age, high sodium intake and African or Hispanic ethnicity [1]. Previous studies have demonstrated that elevated blood pressure (BP) in childhood is highly predictive of hypertension in adulthood [2,3]. Moreover, pediatric hypertension is associated with target organ damage, including left ventricular hypertrophy, increased carotid–intima media thickness and microalbuminuria [2,3]. Nevertheless, several studies have suggested that knowledge, screening, diagnosis, and management for pediatric hypertension is profoundly insufficient among most physicians [4,5].

It has been evident that anthropometrics are related to BP in children, with body mass index (BMI), waist circumference, waist-to-height ratio (WHtR) and skin fold being the most well studied and commonly used parameters [6,7,8]. Parameters of body composition (BC), such as body-fat percentage (BF%) and visceral fat area, have also been reported in many studies to be closely associated with cardiometabolic risk factors [9]. However, studies on the relationships between BF% and visceral fat area and pediatric hypertension are still relatively scarce, and the results have been inconclusive [10,11,12].

We hypothesized that BC parameters could predict the risk of pediatric hypertension. This cross-sectional observational study aimed to investigate the relationships between anthropometrics, BC parameters and BP among school-aged children using data from a school-based health promotion program conducted in northern Taiwan. We further compared the difference in predictive values of pediatric hypertension between anthropometric measures and BC components.

## 2. Materials and Methods

### 2.1. Study Design and Subjects

This study was a quantitative cross-sectional study. We analyzed anonymous data from the database of a school-based health promotion project conducted by a single institution (Chang Gung Memorial Hospital, Linkou Main Branch, Taoyuan) in Taiwan from 2013 to 2016. The project was conducted at several elementary schools in Northern Taiwan for students aged from 7 to 12 years. The details of the project have been reported elsewhere [13]. Most participants were with Han ethnicity. All sensitive information which could be linked to a specific person (name, identity number, class number) were deidentified and stored separately and carefully. Demographic data (age, sex), anthropometric measures, BC parameters, and BP were retrieved for analysis. This study was approved by the Institutional Review Board of our hospital (101-4158A3). Written informed consent was obtained from all participants and their parents.

### 2.2. Anthropometric Measures

The weight (in kg) and height (in cm) of all participants were measured according to standard protocols without shoes. BMI was defined as weight (kg) divided by the height squared (m^2^) [14]. BMI z-scores and percentiles were calculated based on sex and age in months according to the United States Centers for Disease Control and Prevention 2000 growth charts [15]. Waist circumference (in cm) was determined by measuring the circumference in the horizontal plane midway between the lowest ribs and the iliac crest [16]. WHtR was calculated as waist circumference (cm) divided by the height (cm).

### 2.3. Body Composition Parameters

BC parameters were examined using bioelectrical impedance analysis (BIA) (X-scan model, Jawon Medical Co., Daejeon, South Korea). BF%, muscle weight, fat mass, and fat-free mass were automatically obtained. Our BC measurement protocol has been described elsewhere in detail [13].

### 2.4. Blood Pressure Tests and Hypertension Screening

BP was recorded using an automated sphygmomanometer after placing the participant in a seated position for at least 10 min. For those whose BP exceeded the normal range, another 5 min of rest was allowed to recheck the BP, and we recorded the lowest systolic blood pressure (SBP) and diastolic blood pressure (DBP) values after performing the measurements twice. Furthermore, z-scores and percentiles of SBP and DBP were obtained based on sex, height z-score and age in years according to the BP tables [17]. Pediatric hypertension was defined as average clinic SBP and/or DBP ≥ 95th percentile on the basis of age, sex and height percentiles [18].

### 2.5. Statistical Analysis

Statistical analysis was conducted using SPSS software (version 25, International Business Machines Corp., Armonk, NY, USA). A *p*-value < 0.05 was considered to be statistically significant. Continuous variables were summarized as means ± standard deviations and categorical variables were summarized as numbers (percentages). Participants with either an SBP percentile > 95% or DBP percentile > 95% were categorized as being ‘hypertensive’, and those without elevated SBP and DBP were categorized as being ‘normotensive’. Continuous variables were compared using Student’s *t*-tests, and categorical variables were compared using chi-squared tests. Pearson’s correlation test was used to assess relationships between BP measures and continuous variables, and Spearman’s correlation test was used to examine relationships between BP measures and categorical variables or hypertension and variables of interest. Using receiver operator characteristic curves, the optimal cutoff values of anthropometric and BC measures were determined to predict pediatric hypertension using the maximal Youden index [19], and the areas under the curve (AUCs), 95% confidence intervals (CIs), *p*-values, sensitivity, and specificity were calculated. Univariate and multivariate binary logistic regression models were used to assess dichotomized variables and provide odds ratios [20], 95% CIs and *p*-values.

## 3. Results

In total, 340 children (177; 52.1% girls and 163; 47.9% boys) with a mean age of 8.8 ± 1.7 years (range, 7–12 years) were included in the study. Table 1 summarizes the demographic, anthropometric, BC and BP measures in the overall cohort and two subgroups. The hypertensive children had higher anthropometric measures (including BMI, BMI z-score, BMI percentile and WHtR) than the normotensive children. In addition, the hypertensive children had higher BC parameters including BF%, muscle weight, fat mass and fat-free mass than the normotensive children. As expected, BP measures (including SBP, DBP, SBP z-score, DBP z-score, SBP percentile and DBP percentile) of the hypertensive group were higher than those of the normotensive group, even though the distributions of age and sex were comparable between both groups.

Table 2 demonstrates the associations between BP measures and the variables of interest. A higher SBP percentile was associated with all anthropometric measures and BC parameters, and a higher DBP percentile was associated with a higher WHtR and BF%. Pediatric hypertension was associated with all anthropometric measures and BC parameters.

Table 3 summarizes the optimal cutoff values, AUCs, 95% CIs and *p*-values of anthropometric measures and BC parameters for predicting pediatric hypertension. A BMI ≥ 18.75 kg/m^2^, fat mass ≥ 3.65 kg, BMI percentile ≥ 75.5%, BMI z-score ≥ 0.70, BF ≥ 21.35%, fat-free mass ≥ 34.65 kg, WHtR ≥ 0.48 and muscle weight ≥ 32.15 kg, in descending order of AUC, could accurately predict pediatric hypertension.

Table 4 further summarizes the prediction models of pediatric hypertension. Using binary logistic regression models without adjustment, fat-free mass, muscle weight, fat mass, BMI, BF%, BMI percentile, BMI z-score and WHtR—in descending order of OR—were associated with pediatric hypertension. After adjustments for age and sex, fat mass, fat-free mass, muscle weight, BMI, BF%, BMI percentile, BMI z-score and WHtR—in descending order of OR—remained significantly related to pediatric hypertension.

Multivariate binary logistic regression models of all anthropometric variables with forward selection showed that BMI ≥ 18.75 kg/m^2^ independently predicted pediatric hypertension (Table 4). After adjustments for age and sex, BMI ≥ 18.75 kg/m^2^ remained significantly related to pediatric hypertension. Fat mass ≥ 3.65 kg and fat-free mass ≥ 34.65 kg independently predicted hypertension using multivariate analysis of all BC variables (Table 4). After adjustments for age and sex, fat mass ≥ 3.65 kg and fat-free mass ≥ 34.65 kg still independently predicted hypertension. In multivariate analysis including all variables of interest without selection (full model), fat mass ≥ 3.65 kg (OR = 2.21; 95% CI: 1.12–4.37; *p* = 0.022) was the only predictor of pediatric hypertension.

Finally, a combination of fat mass ≥ 3.65 kg and fat-free mass ≥ 34.65 kg was found to be the best prediction model in multivariate logistic regression analysis with forward selection of all variables of interest (AUC = 0.688; 95% CI: 0.627–0.750; *p* < 0.001). With one predictor (either fat mass ≥ 3.65 kg or fat-free mass ≥ 34.65 kg), the sensitivity and specificity to predict pediatric hypertension were 82.4% and 45.4%, respectively. With two predictors (both fat mass ≥ 3.65 kg and fat-free mass ≥ 34.65 kg), the sensitivity and specificity to predict pediatric hypertension were 66.7% and 89.9%, respectively.

## 4. Discussion

The close association between obesity and hypertension is well documented, and many studies have reported the roles of conventional anthropometric measures such as BMI, WHtR and waist circumference in predicting pediatric hypertension [6,12]. In this study, we further examined the associations between BC parameters and BP in children, compared them to conventional measures and identified the best prediction model.

Our results showed that conventional anthropometric measures were all significantly higher in the hypertensive subgroup and also significantly associated with SBP percentile and hypertension. The normal range of BMI increases incrementally with age before adulthood, and the prevalence of hypertension also increases with an older age. Therefore, BMI alone is not sufficient to properly examine the independent relationship between body weight and BP status. On the other hand, BMI z-score and BMI percentile are age-adjusted variables, and WHtR has been shown to be an age-independent parameter. Our results support the positive associations between conventional anthropometric measures and BP in children reported in previous research [6,8]. However, relatively few studies have investigated the use of BC parameters as a screening tool. Skin fold measures and fat distribution indices such as mid-upper-arm circumference, arm-fat area or arm span have been examined in some studies [6,21], however none of them have been demonstrated to be superior to BMI. In our results, all of the four BC parameters (BF%, fat mass, muscle weight and fat-free mass) were higher in the hypertensive subgroup, and they were also all significantly positively correlated with BP measures.

The regulation of BP is intricate and complex to ensure that tissues and organs receive sufficient blood supply for nutrients and oxygen. An elevation in BP can be triggered via an endocrinal response [22,23], nerve reflex [24,25], inflammatory reaction [26,27], volume alteration [28,29] or tissue modification [30,31,32]. The pathologic process implicated in the development and progression of essential hypertension has been extensively described in the literature. It is a slow and progressive process driven by multiple factors. Two main mechanisms are involved in obesity-related hypertension: (1) an expanded plasma volume with an increase in cardiac output and (2) an increase in peripheral vascular resistance [33,34]. Some of these alterations can be a temporary physiological adaptation which may be reversible, while some may be long-term pathologic changes that eventually result in irreversible comorbidities and adverse health outcomes.

An expand in blood volume can be a response to an increased demand for blood supply from skeletal muscles and solid organs [35]. According to Poiseuille’s equation, which describes the behavior of a laminar flow when flowing through pipes:

blood flow (volume/time) = (π∆Pr^4)/8ηλ

(where ∆P = difference in pressure; r = mean radius of the vessel; η = viscosity of the blood; and λ = length of the vessel) [36]. The viscosity of the blood (η) and mean radius of vessels (r) are considered to be nearly the same for children at the same age [37], and total length of blood vessels (λ) is positively correlated with body height. Therefore, the value of (blood pressure)/height ratio in the same age group is likely to be similar given the same required blood flow [14,38]. By definition, muscle weight and fat-free mass are both part of the “lean body mass” of a human body [39], and an increase in lean body mass considerably increases the demand for blood flow, eventually leading to higher BP as a physiological adaptation [35]. This may explain the findings in our investigation that both muscle weight and fat-free mass were independently and positively associated with BP.

On the other hand, adiposity also contributes to an elevation in BP through multiple pathways [33]—mainly dysregulation of renal sodium handling and unfavorable changes in vascular structures. Excessive body fat accumulation causes insulin resistance [40]. Insulin resistance and inflamed adipose tissue cause an increase in adipose-derived angiotensinogen [34] and conversion of angiotensin I to angiotensin II [41]. Taken together, these factors provoke systemic activation of the renin–angiotensin–aldosterone system, leading to increased sympathetic activity, decreased sodium excretion, increased aldosterone production, arteriolar vasoconstriction and anti-diuretic hormone secretion, off all which result in an elevated BP [34]. Adipose tissue also triggers endothelium hyperplasia through an insulin-like growth factor pathway and a decrease in bioavailable nitric oxide [42]. The excessive production of reactive oxygen species related to adipose tissue precipitates chronic systemic inflammation [43], further damaging vascular structures, causing endothelium dysfunction [44,45] and leading to atherosclerosis over time [46]. The cardinal roles that adiposity play in BP elevation may explain our finding of the independent positive associations between BF% and fat mass with pediatric hypertension.

Previous studies report that z-BMI and WHtR are better predictors of pediatric hypertension than skin fold measures, since they present the amount of both lean body mass and fat [47], while skin fold measures can only estimate the peripheral fat of a few isolated parts of the body [6]. However, the fat-related parameters used in the current study, BF% and fat mass, represent both subcutaneous and visceral adiposity. Moreover, the BC model we developed comprised of fat mass > 2.93 and fat-free mass > 2.89 was superior (sensitivity 77.7% and specificity 89.9) to the anthropometric model (sensitivity 55.9% and specificity 73.9). We hypothesize that the better performance of the BC model may be because it evaluated not only the overall weight status of the human body, but also how it was composed, thus more comprehensively and precisely reflecting the different influences on BP from dichotomized body components.

The method applied to examining BC in this study, BIA, has some advantages and disadvantages compared to conventional anthropometric measures in practice. Both two approaches are now widely used in various clinical and community settings, since they require little subject cooperation, and the instruments are portable and can be used almost everywhere. Anthropometrics require more human power to instruct the subject, read the measure and record it, while with BIA most of the steps are done by the machine [39]. Being operator-dependent also makes anthropometrics more likely to have interobserver error, thus in need of more orientation training. However, the BIA devices are still more costly than anthropometrics. In addition, the BC measurement by BIA is very sensitive to hydration status, and the results may not be accurate in individuals with extreme BC, such as athletes, pregnant women or patients with severe illness [48,49].

The main contribution of this study is to provide a novel insight into how BC parameters may be superior to conventional anthropometric measures in predicting pediatric hypertension. This is essential to better understand the risk factors for pediatric hypertension and develop an effective approach for risk stratification. Weight status and body fitness should be screened and managed for children early to prevent comorbidities. A limitation of this investigation is that most of the participants were Han, and this single ethnicity of the cohort may limit its generalizability to other populations. In addition, the reference tables used for BMI [15] and BP [17] were based on surveys from the United States since no equivalently detailed and validated data were available for Han children. In addition, the study was cross-sectional and thus unable to conclude causal relationships. Further studies with a prospective design will be of interest.

## 5. Conclusions

BC parameters are better than conventional anthropometric measures in predicting pediatric hypertension. The inclusion of both fat mass and fat-free mass performed better than BMI alone. Effective risk stratification can allow for more precise and earlier interventions for obesity-related comorbidities such as hypertension in children.

## Figures and Tables

**Table 1:** Demographic, anthropometric, body composition and blood pressure measures of the overall cohort as well as normotensive and hypertensive subgroups.

Variables	Overall	Normotensive Subgroup	Hypertensive Subgroup	*p*-Value ^a^
Patients	*n* = 340	*n* = 238	*n* = 102	
Demographic measures				
Girls (n)	177 (52.1)	126 (52.9)	51 (50.0)	0.62
Age (years)	8.8 (1.7)	8.7 (1.7)	9.0 (1.7)	0.08
Anthropometric measures				
BMI (kg/m^2^)	18.28 (3.67)	17.63 (3.22)	19.78 (4.20)	<0.001
BMI z-score	0.50 (1.24)	0.32 (1.29)	0.93 (0.98)	<0.001
BMI percentile (%)	64.3 (29.3)	59.8 (29.8)	74.9 (25.2)	<0.001
Waist/height ratio	0.47 (0.06)	0.46 (0.06)	0.49 (0.06)	<0.001
Body composition parameters				
Body-fat percentage (%)	16.00 (7.56)	14.85 (7.21)	18.69 (7.70)	<0.001
Fat mass (kg)	5.88 (4.80)	5.02 (3.86)	7.89 (6.07)	<0.001
Muscle weight (kg)	25.58 (7.09)	24.52 (6.18)	28.07 (8.37)	<0.001
Fat-free mass (kg)	28.53 (7.73)	26.35 (6.71)	30.28 (9.16)	<0.001
Blood pressure measures				
SBP (mmHg)	108.5 (14.5)	101.6 (9.3)	124.5 (11.7)	<0.001
DBP (mmHg)	61.1 (11.8)	57.3 (8.7)	69.9 (13.5)	<0.001
SBP z-score	0.72 (1.25)	0.12 (0.77)	2.14 (0.96)	<0.001
DBP z-score	0.11 (1.02)	−0.21 (0.77)	0.84 (1.17)	<0.001
SBP percentile (%)	66.2 (28.4)	54.3 (24.8)	93.9 (12.0)	<0.001
DBP percentile (%)	51.7 (28.6)	43.5 (24.4)	70.6 (28.6)	<0.001

Data summarized as mean (standard deviation) or *n* (%) as appropriate; Abbreviations: BMI—body mass index; DBP—diastolic blood pressure; SBP—systolic blood pressure; ^a^ Data compared using independent Student’s t-test for continuous variables or chi-squared test for categorical variables, as appropriate.

**Table 2:** Correlations ^a^ of blood pressure measures with demographic, anthropometric and body composition measures in the overall cohort.

Variables	SBP Percentile	DBP Percentile	Hypertension
Demographic measures			
Sex (female/male)	0.01 (0.79)	0.04 (0.46)	−0.03 (0.62)
Age (years)	0.11 (0.050)	0.02 (0.73)	0.10 (0.08)
Anthropometric measures			
BMI (kg/m^2^)	0.35 (<0.001)	0.05 (0.39)	0.26 (<0.001)
BMI z-score	0.33 (<0.001)	0.05 (0.38)	0.24 (<0.001)
BMI percentile (%)	0.32 (<0.001)	0.05 (0.39)	0.24 (<0.001)
Waist/height ratio	0.28 (<0.001)	0.11 (0.04)	0.23 (<0.001)
Body composition parameters			
Body-fat percentage (%)	0.34 (0.001)	0.11 (0.04)	0.23 (<0.001)
Fat mass (kg)	0.38 (<0.001)	0.10 (0.06)	0.27 (<0.001)
Muscle weight (kg)	0.27 (<0.001)	0.02 (0.77)	0.20 (<0.001)
Fat-free mass (kg)	0.27 (<0.001)	0.02 (0.75)	0.20 (<0.001)

Data are summarized as *r*-value (*p*-value); Abbreviations: BMI—body mass index; DBP—diastolic blood pressure; SBP—systolic blood pressure; ^a^ Data compared using Pearson’s correlation test for continuous-to-continuous variables or Spearman correlation test for continuous variables-to-categorized variables, as appropriate.

**Table 3:** Single anthropometric measure or body composition parameter as predictors of pediatric hypertension in the overall cohort, in descending order of area under the curve (AUC).

Predictors	Cutoff Value	AUC	95% CI	*p*-Value ^a^
Anthropometric measures				
BMI (kg/m^2^)	18.75	0.649	0.584–0.715	<0.001
BMI percentile (%)	75.5	0.63	0.565–0.694	<0.001
BMI z-score	0.7	0.627	0.562–0.692	<0.001
Waist/height ratio	0.48	0.614	0.547–0.681	0.001
Body composition parameters				
Fat mass (kg)	3.65	0.64	0.578–0.702	<0.001
Body-fat percentage (%)	21.35	0.616	0.548–0.683	0.001
Fat-free mass (kg)	34.65	0.615	0.546–0.683	0.001
Muscle weight (kg)	32.15	0.61	0.541–0.679	0.001

Abbreviations: AUC—area under the receiver operating characteristic curve; BMI—body mass index; CI—confidence interval; DBP—diastolic blood pressure; SBP—systolic blood pressure; ^a^ Data compared using independent Student’s t-test for continuous variables or chi-squared test for categorized variables, as appropriate.

**Table 4:** Prediction models of pediatric hypertension in the overall cohort.

Models	Logistic Regression without Adjustment			Logistic Regression with Adjustments for Age and Sex			Receiver Operator Characteristic Curve (ROC Curve)		
Predictors	Odds Ratio	95% CI	*p*-Value	Odds Ratio	95% CI	*p*-Value	Cutoff Value	Sensitivity	Specificity
**Univariate models** ^a^									
BMI (kg/m^2^)	3.6	2.21–5.85	<0.001	3.53	2.12–5.89	<0.001	≥18.75	55.90%	73.90%
BMI z-score ^b^	2.82	1.75–4.56	<0.001	–			≥0.70	62.70%	62.60%
BMI percentile ^b^	2.89	1.79–4.67	<0.001	–			≥75.5	63.70%	62.20%
Waist/height ratio	2.74	1.69–4.46	<0.001	2.82	1.72–4.63	<0.001	≥0.48	48.00%	74.80%
Body-fat percentage	3.17	1.90–5.31	<0.001	3.05	2.03–6.06	<0.001	≥21.35	41.20%	81.90%
Fat mass	3.82	2.18–6.68	<0.001	5.01	2.66–9.43	<0.001	≥3.65	81.40%	46.60%
Muscle weight	3.91	2.20–6.94	<0.001	4.26	2.19–8.31	<0.001	≥32.15	66.70%	88.70%
Fat-free mass	4.08	2.30–7.24	<0.001	4.54	2.33–8.84	<0.001	≥34.65	34.30%	88.70%
**Anthropometric Model** ^c^							≥1	55.90%	73.90%
BMI ≥ 18.75 kg/m^2^	3.6	2.21–5.85	<0.001	3.53	2.12–5.89	<0.001			
**Body Composition Model** ^d^							≥2	66.70%	89.90%
Fat mass ≥ 3.65 kg	2.93	1.64–5.26	<0.001	3.84	2.00–7.39	<0.001			
Fat-free mass ≥ 34.65 kg	2.89	1.59–5.26	<0.001	2.98	1.48–5.99	0.002			

Abbreviations: AUC—area under the curve; BMI—body mass index; CI—confidence interval; DBP—diastolic blood pressure; SBP—systolic blood pressure; ^a^ Single anthropometric or body composition variables were analyzed using binary logistic regression models; ^b^ BMI z-score and BMI percentile adjusted for age and sex at baseline; ^c^ Four anthropometric variables included for multivariate binary logistic regression models with forward selections; ^d^ Four body composition variables included for multivariate binary logistic regression models with forward selection.
